# Early and mid-term outcome of frozen elephant trunk using spinal cord protective perfusion strategy for acute type A aortic dissection

**DOI:** 10.1007/s11748-020-01328-z

**Published:** 2020-03-09

**Authors:** Yu Hohri, Takuma Yamasaki, Yuichi Matsuzaki, Takeshi Hiramatsu

**Affiliations:** Department of Cardiovascular Surgery, Japanese Red Cross Kyoto Daini Hospital, 355-5 Haruobi-cho, Kamigyo-ku, Kyoto, 602-8026 Japan

**Keywords:** Frozen elephant trunk, Total arch replacement, Acute aortic dissection, Spinal cord injury, Aortic balloon occlusion

## Abstract

**Objective:**

This study aimed to evaluate the prevalence of spinal cord injury in total arch replacement with frozen elephant trunk for acute type A aortic dissection using our spinal cord protection technique.

**Methods:**

Between January 2013 and December 2017, 33 patients underwent total arch replacement with frozen elephant trunk for acute type A aortic dissection (mean age 67.9 ± 13.3 years). Our spinal cord protection technique involved maintaining extracorporeal circulation through the left subclavian artery in all procedures, using aortic occlusion balloon during distal anastomosis, and inserting frozen elephant trunk above Th 8 with transesophageal echocardiographic guidance. Computed tomography was performed within 1–2 weeks, 12 months, and 36 months postoperatively. We compared the degree of thrombosis of the descending aorta between preoperation and early postoperative period by Fisher’s exact test. Moreover, we evaluated postoperative mortality and mobility (including spinal cord injury) at follow-up.

**Results:**

The operative mortality within 30 days was 6.1%. Neither paraplegia nor paraparesis was noted. We observed significant thrombosis of the false lumen at the distal arch and aortic valve level of the descending aorta in postoperative early term period (*p* < 0.01). At mid-term follow-up (mean 33.9 months), survival probability and 3-year freedom from reoperation rates were 93.9 ± 4.1% and 95.0 ± 4.9%, respectively.

**Conclusions:**

The frozen elephant trunk technique with our spinal protection strategy provides good postoperative outcomes. Our strategy can maintain spinal cord perfusion without complete ischemia time even during lower body ischemia time. Implementation of our spinal protection strategy will help prevent spinal cord injury and dilated downstream aorta.

## Introduction

Hemiarch replacement (HAR) or ascending aorta replacement has been the most commonly performed surgical procedure for acute type A aortic dissection (AAAD) [1–3]. However, because this approach leaves the downstream aorta untouched, a patent false lumen promotes enlargement of the downstream aorta, increasing the risk of aortic rupture and reoperation [4–6]. A hybrid surgical approach, known as the total arch replacement with frozen elephant trunk (TAR-FET) technique, has recently received considerable interest. Some studies have reported that TAR-FET can prevent the dilation of the dissecting aneurysms [7–9]. However, TAR-FET for AAAD could be complicated with spinal cord injury (SCI) [10–12]. SCI is one of the most devastating complications of TAR-FET [13], and survival rates among patients with paraplegia are significantly lower than those among the able-bodied patients [14, 15].

A previous study reported that the SCI showed moderate positive correlation with circulatory arrest time [16], so we consider that it is one of the most important methods to make the spinal cord ischemia time as short as possible. We devised a method of continued spinal perfusion without complete ischemia time of the spinal cord in TAR-FET. Furthermore, we permit the distal landing zone until Th 8 to promote aortic remodeling and to prevent dilatation of the descending aorta at postoperative follow-up period. This study mainly aimed to present our strategy for SCI prevention with TAR-FET and then to report the early and mid-term outcomes of this procedure.

### Subjects

Between January 2013 and December 2017, 66 patients underwent surgical treatment for AAAD at the Japanese Red Cross Kyoto Daini Hospital in Japan. Previously, we performed HAR as the salvage procedure for AAAD; however, TAR-FET has been commonly performed to avoid progressive dilation of the dissecting aneurysm after J Graft Open Stent Graft (Japan Lifeline, Tokyo, Japan) received commercial approval in Japan. Among these patients, 33 patients underwent TAR-FET. They were identified using contrast-enhanced computed tomography (CT) (Aquilion Prime 80 series; Canon Medical Systems, Tokyo, Japan). We included only patients with acute aortic dissection with symptoms, such as chest pain and backache and excluded those with chronic aortic dissection and accidently detected aortic dissection, as their onset was not clear. Eight patients were not eligible for FET implantation based on the following exclusion criteria: very elderly status (age > 85 years), DeBakey classification type II, and inability to receive extended anticoagulant treatment because of risk of hemorrhagic cerebral infarction.

## Methods

The chief surgeon (Takuma Yamasaki) performed the surgery in all patients. All procedures involved implantation of a J Graft Open Stent Graft or J Graft FROZENIX (Japan Lifeline, Tokyo, Japan) into the descending aorta. The primary entry tear was observed with preoperative contrast-enhanced CT or intraoperative visual inspection. Our hospital’s ethics committee approved this retrospective study; informed consent was obtained from patients prior to surgery. Moreover, this study was conducted in accordance with the principles outlined in the Declaration of Helsinki and all its provisions.

### TAR-FET procedure

**Fig. 1:**
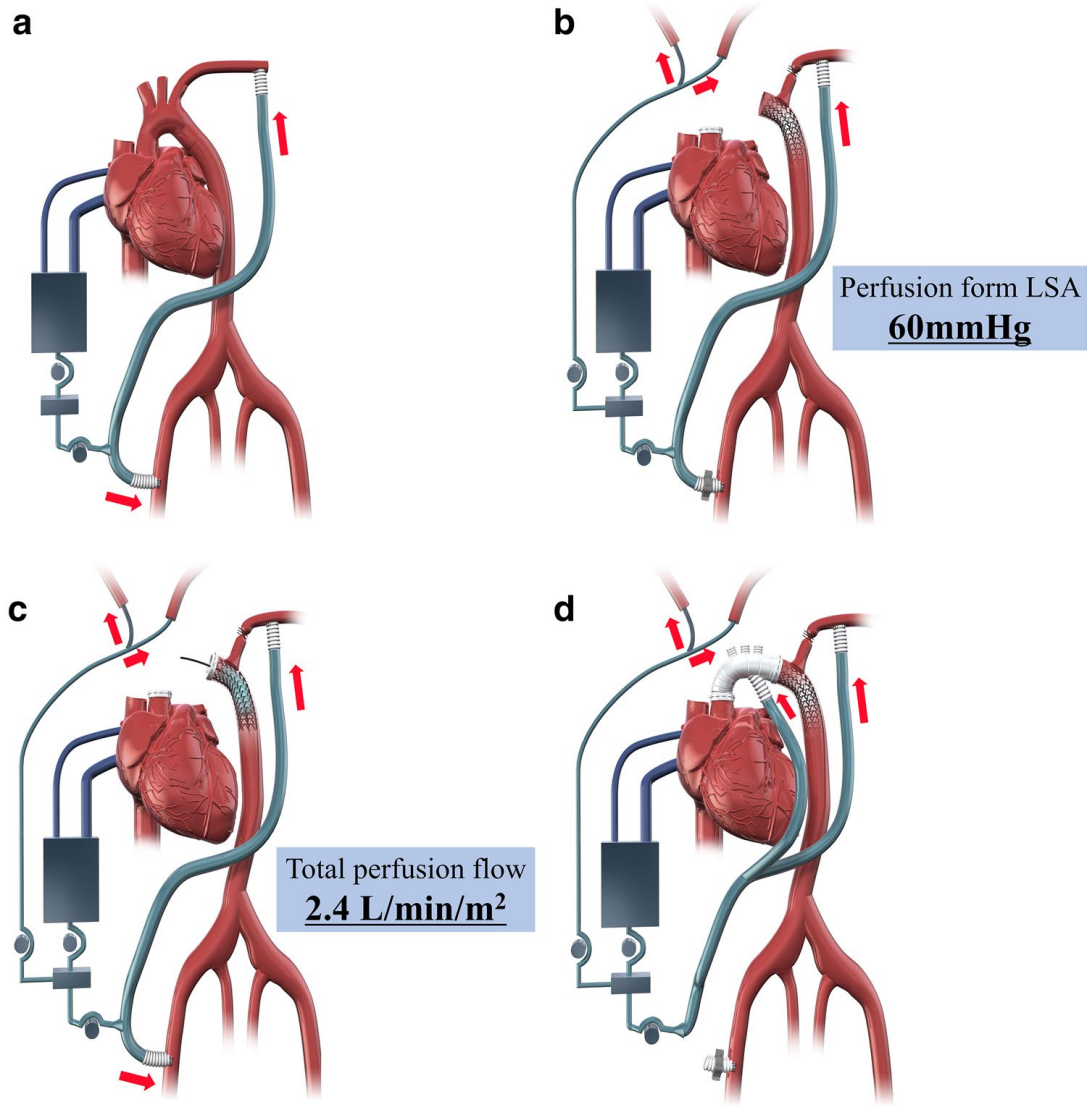
Extracorporeal circulation during TAR-FET. **a** Two arterial perfusion cannulas are inserted into the left subclavian and unilateral femoral arteries and anastomosed to a 9-mm synthetic graft. A two-stage venous cannula is inserted through the right atrium, and a left ventricular vent tube is inserted through the right upper pulmonary vein. **b** When the urinary bladder temperature decreases to 28 °C, the left subclavian artery is ligated and the perfusion pressure of the left subclavian artery is maintained at to average of 60 mmHg under lower body circulatory arrest. After the aortic arch is incised, the balloon catheter is inserted into the brachiocephalic and left carotid arteries, and selective cerebral perfusion is established. The FET is deployed under transesophageal echocardiographic guidance. **c** After the FET is inserted, an occlusion balloon is inserted into it, and the lower body circulation is restarted from the femoral artery. The total perfusion flow rate is 2.4 L/min/m^2^. **d** After distal anastomosis, antegrade perfusion is restarted and proximal anastomosis is performed. *TAR-FET* total arch replacement with frozen elephant trunk, *LSA* left subclavian artery

**Fig. 2:**
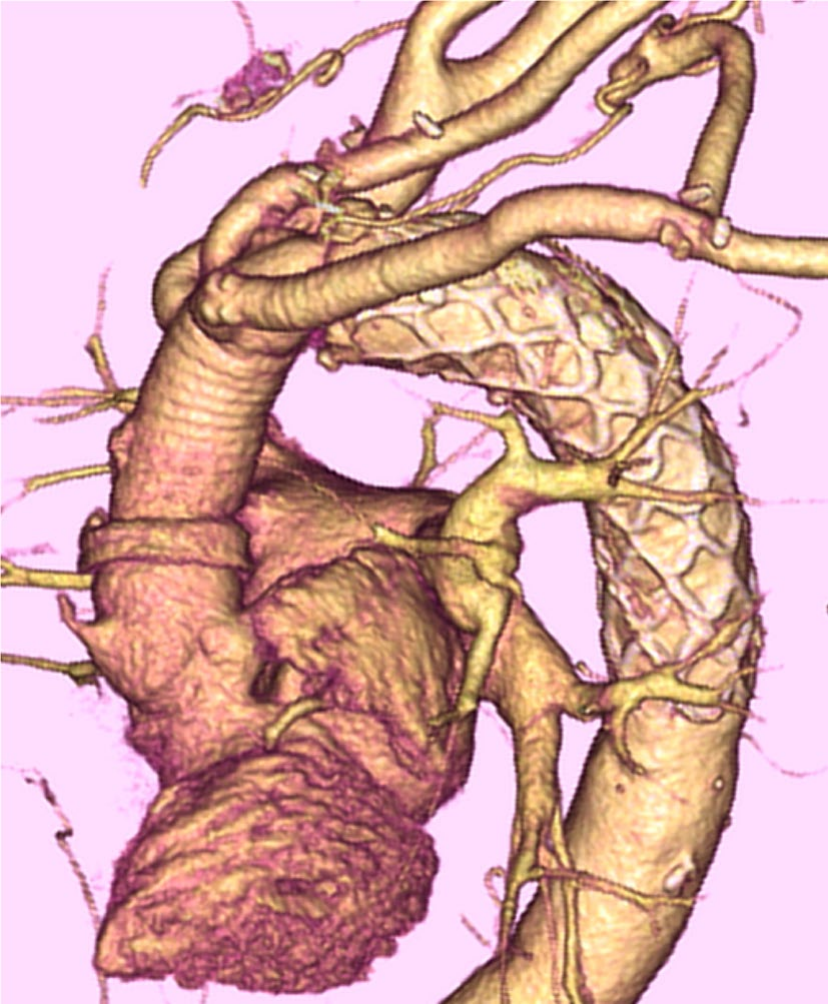
Postoperative computed tomography imaging in total arch replacement with frozen elephant trunk using our strategy

Extracorporeal circulation during TAR-FET is shown in Fig. 1. A median sternotomy was employed for all TAR-FET procedures. Two arterial perfusion cannulas were inserted into the left subclavian and unilateral femoral arteries anastomosed to a 9-mm synthetic graft. A right subclavian artery cannula was additionally inserted in patients with right cerebral malperfusion. A two-stage venous cannula was inserted through the right atrium, and a left ventricular vent tube was inserted through the right upper pulmonary vein (Fig. 1a). The ascending aorta was clamped and incised. The proximal aortic stump was covered with inner and outer felt strips using 4-0 polypropylene horizontal mattress sutures. After the urinary bladder temperature decreased to 28 °C, the left subclavian artery was ligated, and the perfusion pressure of the left subclavian artery was maintained at an average of 60 mmHg under lower body circulatory arrest. The aortic arch was incised, the balloon catheter was inserted into the brachiocephalic and left carotid arteries, and selective cerebral perfusion was established at a rate of 1.2 L/min/m^2^. The FET was deployed under transesophageal echocardiographic (TEE) (Philips North America, LLC, Andover, MA, USA) guidance, with the distal segment maintained at almost the same level as the aortic valve (Fig. 1b). After the FET was inserted, an occlusion balloon (12 Fr; LeMaitre Vascular INC., Burlington, MA, USA) was immediately inserted into it, and lower body circulation was restarted from the femoral artery. The total perfusion flow (including selective cerebral perfusion and lower body circulation) rate was 2.4 L/min/m^2^. The distal aortic stump was wrapped from the inside to the outside with the proximal segment of the synthetic stent graft using continuous suture with 4-0 polypropylene (Fig. 1c). Distal anastomosis of the four-branched graft to the native aorta was performed using 3-0 polypropylene suture between the left carotid artery and left subclavian artery (zone 2). Antegrade perfusion was restarted, and proximal anastomosis was performed in the same manner (Fig. 1d). After the release of the aortic clamp, the brachiocephalic and left carotid arteries were anastomosed to the four-branched graft with 4-0 polypropylene suture. Finally, the 9-mm synthetic graft anastomosed to the left subclavian artery was guided into the mediastinum and anastomosed to the four-branched graft to complete the procedure (Fig. 2).

In this study, we always selected the size of the FET based on the aortic diameter at the distal landing site of the descending aorta. If the true lumen was preserved, a size 2–3 mm larger than the diameter measured from the circumference of the true lumen at the distal landing site of the descending aorta was chosen. If the true lumen was displaced into a false cavity and the exact diameter could not be determined, a size of 90% of the total aortic diameter at the distal landing site of the descending aorta was selected. This is because, as reported by Katayama, the total aortic diameter increases by 8% because of aortic dissection [7].

### Spinal cord protection strategy in TAR-FET technique

**Fig. 3:**
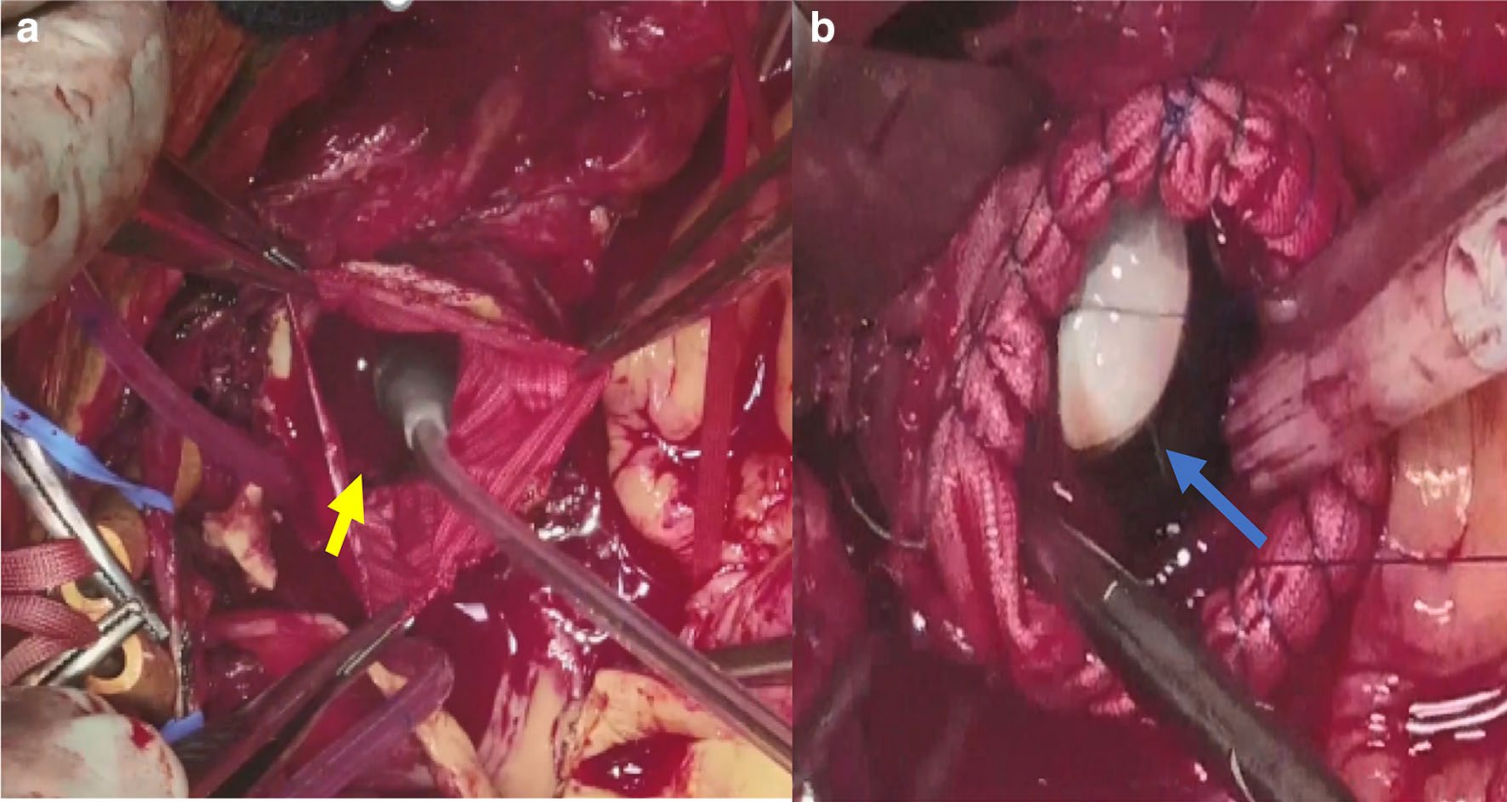
Aortic balloon technique for prevention of spinal cord injury. **a** After a frozen elephant trunk is inserted into the descending aorta, we insert an aortic occlusion balloon (yellow arrow). **b** The aortic occlusion balloon (blue arrow) is inflated, and lower body circulation is immediately restarted. With regard to the distal aortic stump, the proximal segment of the synthetic stent graft is wrapped from the inside to the outside and continuous suturing is performed with 4-0 polypropylene

Several novel techniques were developed to prevent SCI in the TAR-FET procedure. First, we maintained arterial perfusion through the left subclavian artery and evaluated the arterial pressure in the left radial artery as an indicator of perfusion pressure through the left subclavian artery. The arterial pressure was maintained at 60 mmHg even during lower body circulatory arrest. Second, to make the spinal cord ischemia time as short as possible, we used an aortic occlusion balloon immediately after inserting the FET and re-started lower body circulation while performing distal anastomosis (Fig. 3a, b). Third, the FET was deployed under TEE guidance at Th 8 or above to prevent the stent graft from disrupting the atheromatous plaque at the distal landing zone, following the previous report by Katayama et al. [17].

### Follow-up

Postoperative contrast-enhanced CT was routinely performed within 1–2 weeks. We evaluated the degree of thrombosis of the false lumen at each level (distal arch, aortic valve, and celiac artery level). The presence of complete thrombosis of the false lumen that was not enhanced during the arterial phase of CT was documented as thrombosis, while incomplete thrombosis (including partial thrombosis and complete patency) was documented as patency. Enhanced or nonenhanced CT was performed 12 and 36 months postoperatively. The use of contrast enhancement was decided according to each patient’s characteristics (e.g., chronic kidney disease). We evaluated the aortic diameter at the aortic valve and celiac artery levels　of the descending aorta at preoperation, 1–2 weeks, 12 months, and 36 months postoperatively.

### Statistical analysis

All statistical analyses were performed using JMP software version 13.2.0. (SAS Institute, Cary, NC). All data were retrospectively analyzed. Continuous data (operation time, cardiopulmonary time, cardiac arrest time, lower body ischemia time) are expressed as mean ± standard deviation. Categorical data (preoperative characteristics, concomitant surgery, length and diameter of stent graft, distal landing position, and postoperative outcome) are expressed as numbers and percentages. Survival probability and freedom from reoperation on the dilated downstream aorta were estimated using the Kaplan–Meier method. The number of patients with patent false lumen of the descending aorta was analyzed using Fisher’s exact test.

## Results

### Patient characteristics and operative data

Table 1 shows the patients’ clinical characteristics and operative data. With regard to operative data, four patients had cardiac pulmonary arrest (CPA) preoperatively, of which three were alive when they were brought to our hospital and had short arrest time and one developed AAAD during hospitalization for gastritis. The mean lower body ischemia time was 19.2 ± 5.9 min (range 2–34 min), and there was no complete circulatory arrest time in all cases. The average overall diameter of the stent grafts was 28.9 ± 3.3 mm (range 23–35 mm). We used 90-mm or 120-mm stent grafts in all patients (39.4% and 60.6%, respectively), and no patient received a 60-mm stent graft. The average position of the distal edge of the stent graft was Th 7.1 ± 1.0.

**Table 1:** Preoperative characteristics and operative data

	TAR-FET (*n* = 33)
Preoperative characteristics	
Age (years)	67.8 ± 13.2
Sex (male, %)	19 (57.6%)
Hypertension	21 (63.6%)
Diabetes mellitus	3 (9.1%)
Creatinine > 2 mg/dL	2 (6.1%)
History of cerebrovascular event	1 (3.0%)
Preoperative CPA	4 (12.1%)
Tamponade	7 (21.2%)
Site of entry tear	
Ascending aorta	12 (36.4%)
Aortic arch	20 (60.6%)
Descending aorta	1 (3.0%)
Marfan syndrome	0 (0.0%)
Malperfusion	
Cerebral	11 (33.3%)
Cardiac	2 (6.1%)
Intestinal	0 (0.0%)
Renal	3 (9.1%)
Lower limb	3 (9.1%)
Operative data	
Operation time (min)	361.3 ± 62.7
Cardiopulmonary time (min)	192.4 ± 53.0
Cardiac arrest time (min)	119.9 ± 24.4
Lower body ischemia time (min)	19.2 ± 5.9
Concomitant surgery	
CABG	0 (0.0%)
AVR or AVP	5 (15.2%)
Bentall	0 (0.0%)
Axillo-femoral bypass	1 (3.0%)
Frozen elephant trunk	
External diameter of stent graft	
23 mm	2 (6.1%)
25 mm	5 (15.2%)
27 mm	5 (15.2%)
29 mm	10 (30.3%)
31 mm	4 (12.1%)
33 mm	5 (15.2%)
35 mm	2 (6.1%)
Length of stent graft	
60 mm	0
90 mm	13 (39.4%)
120 mm	20 (60.6%)
Distal landing position	
Th 6	8 (24.2%)
Th 7	15 (45.5%)
Th 8	8 (24.2%)
Th 9	0
Th 10	2 (6.1%)

*AVP* aortic valve repair, *AVR* aortic valve replacement, *CABG* coronary artery bypass grafting, *CPA* cardiac pulmonary arrest, *TAR-FET* total arch replacement with frozen elephant trunk, *Th* thoracic vertebrae

### Early and mid-term results

Table 2 presents early and mid-term results. Overall, two patients died within 30 days of the operation because of preoperative severe cerebral malperfusion and CPA. In our study, no SCIs, including paraplegia and paraparesis, were observed. Thoracic endovascular aortic repair (TEVAR) was performed as a planned staged hybrid approach within 1 month postoperatively in two patients. A patient underwent TEVAR for excessive distal insertion of the stent graft leading to a distal landing at Th 10. The proximal synthetic graft was kinked at the distal arch, requiring TEVAR to dilate the proximal synthetic graft. Another patient underwent TEVAR because of aberrant left subclavian artery. During operation, the left subclavian artery could not be ligated; as a result, secondary surgery was planned for closure of the ostium of the aberrant left subclavian artery. Cerebrovascular events occurred in six patients, all of whom were in a critical preoperative condition, including preoperative CPA in four patients and deep coma owing to severe preoperative cerebral malperfusion in two patients.

**Table 2:** Early-and mid-term results

	TAR-FET (*n* = 33)
Early results	
Early mortality	2 (6.1%)
Preoperative CPA	1 (3.1%)
LOS (severe cardiac malperfusion)	0 (0.0%)
Severe cerebral malperfusion	1 (3.1%)
Early reoperation (planned staged operation)	2 (6.1%)
Early morbidities	
LOS	0 (0.0%)
Cerebrovascular event	6 (18.2%)
Spinal cord injury	0 (0.0%)
Respiratory complication	2 (6.1%)
Mediastinitis	0 (0.0%)
Sepsis	0 (0.0%)
Malperfusion	
Cerebral	6 (18.2%)
Cardiac	0 (0.0%)
Intestinal	0 (0.0%)
Renal	0 (0.0%)
Lower limb	1 (3.0%)
Mid-term results	
Mortality	0 (0.0%)
Aortic event	3 (9.1%)
Reoperation for dilated down stream	1 (3.1%)

*CPA* cardiac pulmonary arrest, *LOS* low output syndrome, *TAR-FET* total arch replacement with frozen elephant trunk

**Fig. 4:**
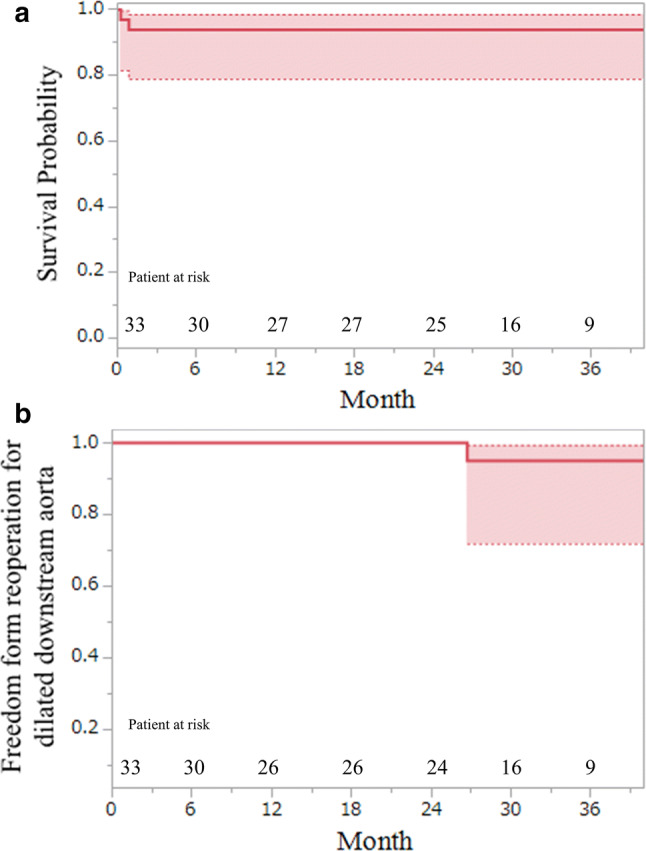
Kaplan–Meier estimate of survival probability and freedom from reoperation on the downstream aorta in TAR-FET. **a** Survival probability in the TAR-FET. **b** Freedom from reoperation on the dilated downstream aorta in the TAR-FET. *TAR-FET* total arch replacement with frozen elephant trunk

Mid-term results were available for 31 patients, and the mean follow-up period was 33.9 ± 21.0 months. Mid-term mortality (excluding 30 days mortality) was not recorded, and the survival rate at 3 years was 93.9 ± 4.1% (Fig. 4a). Aortic events occurred in three patients who had aortic root dilatation, acute type B aortic dissection, or downstream dilatation. Consequently, the reoperation for downstream aorta dilation was required in one patient, and the freedom from reoperation rate at 3 years was 95.0 ± 4.9% (Fig. 4b).

### Aortic remodeling

**Fig. 5:**
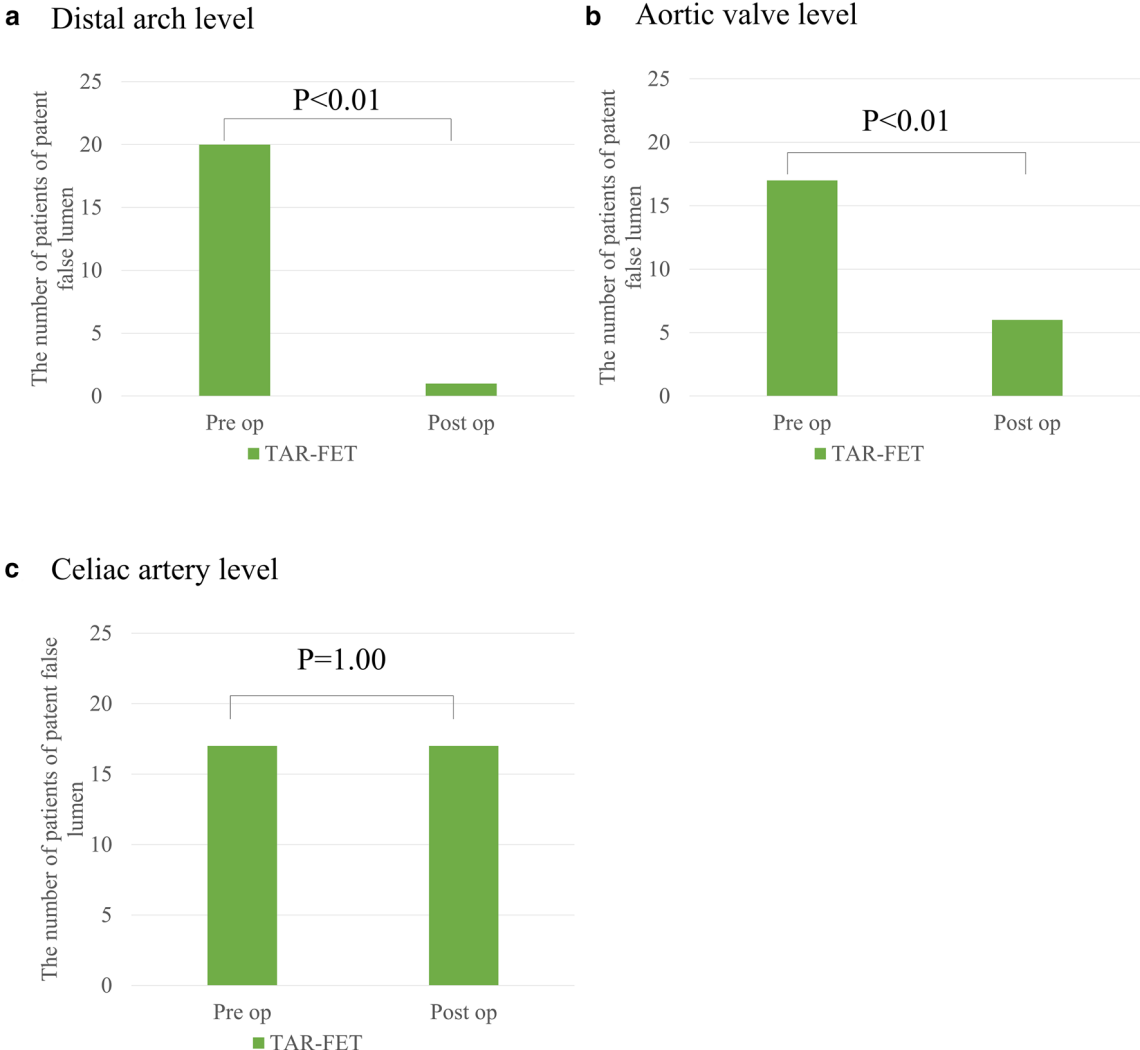
Changes in the patency of the false lumen in the downstream aorta at the distal arch, aortic valve, and celiac artery levels. The number of patients with patent false lumens at the distal arch and the aortic valve level is significantly decreased in the TAR-FET group (*p* < 0.01) by Fisher’s exact test. At the celiac artery level, no significant change is observed. *TAR-FET* total arch replacement with frozen elephant trunk

**Fig. 6:**
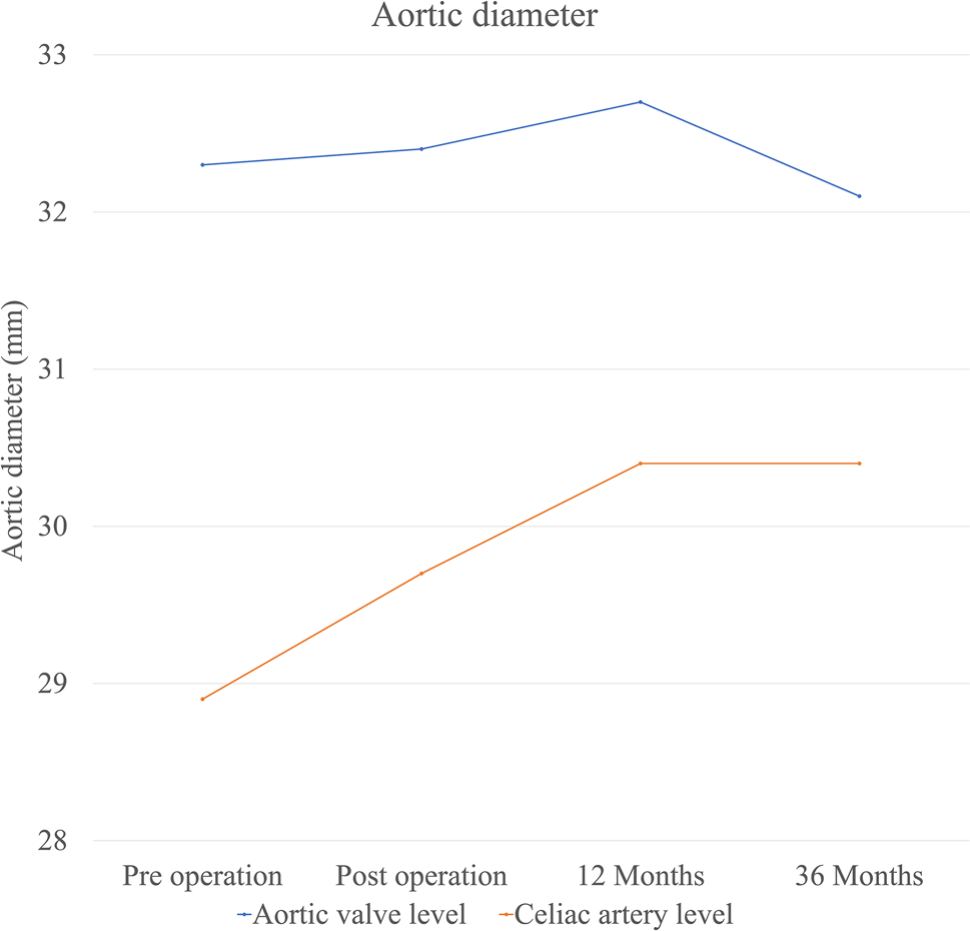
Changes in downstream aortic diameter at the aortic value and celiac artery levels from preoperation levels to 36 months postoperation. At the aortic valve level, the aortic diameter is not more dilated in the TAR-FET. At the celiac artery level, the aortic diameter gradually increases. *TAR-FET* total arch replacement with frozen elephant trunk

A patent false lumen was preoperatively identified in 20 patients at the distal arch and in 17 patients at the aortic valve and celiac artery levels. In early postoperative period, a patent false lumen was observed in the distal arch in one patient and at the aortic valve and celiac artery levels in 6 and 17 patients, respectively (Fig. 5). The aortic diameter of the descending aorta did not change at the aortic valve level; however, it increased gradually at the celiac artery level (Fig. 6).

## Discussion

In this study, we revealed that the surgical strategy in TAR-FET with spinal cord protection techniques for AAAD reduces in-hospital mortality to below the annual level reported by the Japanese Association for Thoracic Surgery (6.1% vs 9.4%) [1]. Moreover, we prevented SCI in all analyzed cases. Our spinal cord protection strategy involved maintenance of extracorporeal circulation through the left subclavian artery, use of an aortic occlusion balloon to preserve spinal cord perfusion during operations, and TEE guidance during FET insertion to prevent thromboembolism to the spinal cord in the presence of severe atherosclerosis at the distal landing zone of the stent graft [18]. In particular, the aortic perfusion maintenance is recommended as spinal cord protection in previous studies [19]. In our study, the aortic occlusion balloon technique and maintenance of arterial perfusion through the left subclavian artery were typical and essential techniques to continue spinal cord perfusion without complete ischemia time. Furthermore, our surgical strategy with TAR-FET resulted in complete thrombosis of the false lumen and prevented re-operation for dilated downstream aorta in many cases. Therefore, this study suggests that our spinal cord protection strategy is effective in preventing SCI and improving postoperative outcomes in the early and mid-term period associated with TAR-FET.

### Spinal cord protection with TAR-FET

We suggest that spinal cord perfusion through the intercostal arteries and collateral networks should be preserved throughout the procedure without interruption for prevention of SCI. In a previous meta-analysis, SCI occurred in 3.1% of patients who underwent TEVAR for complicated acute type B aortic dissection (ABAD) [20]. This rate is lower than the rate of SCI in TAR-FET for AAAD; nevertheless, TEVAR for ABAD also involves occupying the intercostal artery by the stent graft by itself or the thrombosed false lumen like TAR-FET for AAAD. Therefore, we suppose that the hemodynamics during the operation can be a risk factor for SCI, in addition to the thrombosed false lumen and stent graft by itself. Spinal cord perfusion was reported to depend on the spinal arterial blood pressure. Moreover, because a previous study reported that nonperfusion from the left subclavian artery is a risk factor for SCI in TAR-FET [21], it is important to preserve the blood flow in the collateral network to the spinal cord. Thus, arterial blood perfusion during operation is an important factor for the prevention of SCI. With the use of the aortic occlusion balloon, the lower body ischemia time was remarkably short, and spinal cord perfusion through the intercostal arteries could be preserved with the exception of the lower body circulatory arrest period. Occlusion with aortic balloon is a safe technique because we inflate this soft balloon with direct visualization on the FET and not the dissecting aorta directly. Moreover, we measure the volume of saline to dilate the aortic balloon to the same size as the FET before insertion of FET, and never use larger volume of saline than those measured: we do not allow maximum inflation because maximum dilatation of the balloon carries the risk of injuring the intima of the aortic wall. In addition, we used the left subclavian artery as the arterial cannulation site for cardiopulmonary bypass. The perfusion in the cardiopulmonary bypass from the left subclavian artery helps maintain spinal cord perfusion through the vertebral arteries and other branches of the subclavian artery even during the lower body circulatory arrest period.

Thus, the combination of aortic balloon occlusion and circulation from the left subclavian artery enabled continuous spinal cord perfusion without complete ischemia time in TAR-FET. In addition to patients included in this study, we performed TAR-FET for thoracic aortic aneurysms and acute or chronic type B aortic dissections in 90 patients without postoperative SCI [22]. A previous systematic review showed that SCI occurred in 5.1% of patients who underwent TAR-FET (range 0–24%), and the weighted average circulatory arrest time was 48 ± 24 min [16]. Compared with this review, our lower body ischemia time was much shorter (mean 19.2 ± 5.9 min; range 2–34 min), with no complete ischemia of the spinal cord. Therefore, our results support the importance of maintaining spinal cord perfusion in preventing SCI.

### Aortic remodeling with FET technique for AAAD

In this study, the false lumen could be thrombosed significantly at the aortic valve level of the descending aorta postoperatively. We assumed that the false lumen of the descending aorta at the aortic valve level could be thrombosed and could achieve excellent aortic remodeling because we used long stent grafts (only 90-mm or 120-mm stent grafts). Despite some studies reporting elevated SCI risk with a distal landing zone of Th 7 or greater [18, 23], because we used continuous spinal cord perfusion to decrease SCI risk, we were able to safely deploy the FET to Th 8. FET can prompt the false lumen to be thrombosed at the descending aorta because the stent graft helps close the primary entry, and at the mid-term, the true lumen of the descending aorta expanded because of the radial forces exerted by stent graft expansion and the false lumen degenerated with aortic remodeling. In previous studies, using long stent grafts, the false lumen was thrombosed effectively, and the diameter of the downstream aorta could prevent enlargement in the postoperative period [24]. In this study, we used only 90-mm or 120-mm FET (never 60 mm); consequently, the descending aorta, even at the aortic valve level, thrombosed during the early period, preventing enlargement during the follow-up period. Moreover, 13 patients who may have had thrombosed false lumen at the descending aorta were included in this study. These patients can also gain good aortic remodeling by the radial force with stent graft expansion. Furthermore, even if the false lumen is thrombosed at the descending aorta, there are usually some micro re-entries in the descending aorta. Thus, the FET can occupy these re-entries and prevent postoperative aortic dilation by converting these micro re-entries into a new primary entry. In fact, at our institution, 19 patients who had thrombosed false lumen of the descending aorta underwent HAR during the same period. The aortic diameter did not change in patients with TAR-FET but was increased in those with HAR (− 0.6 mm/3 years vs. 2.7 mm/3 years).

In contrast, the aortic diameter at the celiac artery level gradually increased because of the persistence of the abdominal re-entry postoperatively, which preserved the blood flow and precluded the thrombosis of the abdominal aortic false lumen. However, no patient required reoperation for dilated aneurysms at the celiac artery level because of the slow rate of dilation.

## Limitations

Our study has several limitations. First, the study had a retrospective design and focused on analyzing surgical outcomes. Second, the follow-up period was not long enough to determine long-term changes in the descending aorta. Thus, it is necessary to consider a longer clinical follow-up for all patients who underwent this hybrid procedure. Third, the number of patients was too small to conclude the effectiveness of TAR with the FET technique. Finally, all procedures in our study were performed at a single center by a single surgeon, thereby introducing the possibility of selection bias. Thus, we must increase the number of patients and expand the study to multiple centers in different regions in a future study.

## Conclusion

TAR-FET for AAAD with our spinal cord protection strategy did not result in SCI occurrence and achieved excellent aortic remodeling. Our surgical strategy contains two typical techniques for the prevention of SCI. First, the extracorporeal circulation through the left subclavian artery is maintained even during lower body ischemia time. Second, the aortic occlusion balloon is inserted into the FET during the distal anastomosis to preserve spinal cord perfusion through the intercostal arteries. Implementation of our spinal protection strategy during TAR-FET may help prevent SCI and dilated downstream aorta associated with the TAR-FET procedure.
